# Corticosterone and Glucocorticoid Receptor in the Cortex of Rats during Aging—The Effects of Long-Term Food Restriction

**DOI:** 10.3390/nu13124526

**Published:** 2021-12-17

**Authors:** Vesna Tesic, Jelena Ciric, Irena Jovanovic Macura, Nevena Zogovic, Desanka Milanovic, Selma Kanazir, Milka Perovic

**Affiliations:** 1 Department of Neurobiology, Institute for Biological Research “Sinisa Stankovic”—National Institute of Republic of Serbia, University of Belgrade, Bul. despota Stefana 142, 11060 Belgrade, Serbia; vesna.tesic@ibiss.bg.ac.rs (V.T.); jelena.ciric@ibiss.bg.ac.rs (J.C.); irena.macura@ibiss.bg.ac.rs (I.J.M.); desan@ibiss.bg.ac.rs (D.M.); milkap@ibiss.bg.ac.rs (M.P.); 2 Department of Neurophysiology, Institute for Biological Research “Sinisa Stankovic”—National Institute of Republic of Serbia, University of Belgrade, Bul. despota Stefana 142, 11060 Belgrade, Serbia; nevenar@ibiss.bg.ac.rs

**Keywords:** food restriction, intermittent fasting, glucocorticoids, GRα isoforms, 11β-HSD1, c-fos

## Abstract

Numerous beneficial effects of food restriction on aging and age-related pathologies are well documented. It is also well-established that both short- and long-term food restriction regimens induce elevated circulating levels of glucocorticoids, stress-induced hormones produced by adrenal glands that can also exert deleterious effects on the brain. In the present study, we examined the effect of long-term food restriction on the glucocorticoid hormone/glucocorticoid receptor (GR) system in the cortex during aging, in 18- and 24-month-old rats. Corticosterone level was increased in the cortex of aged ad libitum-fed rats. Food restriction induced its further increase, accompanied with an increase in the level of 11β-hydroxysteroid dehydrogenase type 1. However, alterations in the level of GR phosphorylated at Ser^232^ were not detected in animals on food restriction, in line with unaltered CDK5 level, the decrease of Hsp90, and an increase in a negative regulator of GR function, FKBP51. Moreover, our data revealed that reduced food intake prevented age-related increase in the levels of NFκB, *gfap*, and *bax*, confirming its anti-inflammatory and anti-apoptotic effects. Along with an increase in the levels of *c-fos*, our study provides additional evidences that food restriction affects cortical responsiveness to glucocorticoids during aging.

## 1. Introduction

Moderate reduction in food intake [food restriction (FR)] is widely accepted as one of the few effective approaches to extend lifespan, slow-down physiological aging, and to delay the onset or reduce the severity of age-associated diseases [1,2]. Numerous anti-aging effects of FR have also been reported for the brain, including improvements in synaptic integrity [3,4,5,6], motor, and to some extent cognitive performances [7,8,9,10]. In particular, although negative effects are rarely reported, some inconsistency remains regarding the effects of FR on cognitive functions, including age-related cognitive decline (reviewed in [11,12]).

Among specific cellular and molecular mechanisms that may account for beneficial anti-aging and neuroprotective effects of FR, the most extensively reported are the decrease in metabolic rate, increase in insulin sensitivity, and those resulting in promoting anti-oxidative and anti-inflammatory capacity [1]. Moreover, both acute and long-term FR regimens are known to induce moderate elevation of glucocorticoids in the blood [13,14,15]. It has been therefore proposed that FR functions as a mild stressor that stimulates the production of stress resistance proteins important for coping with stronger stressors [16]. Qiu and colleagues [17] reported, however, that FR-induced neuroprotection in rats is further enhanced by the reduction of glucocorticoids levels. This is in line with well-established detrimental effects of chronic exposure to excess glucocorticoids on the brain, which were further associated with learning and memory impairments, and various pathologies including age-related neurodegenerative diseases, dementia, and psychosis [18,19,20,21].

Glucocorticoids (cortisol in primates and corticosterone in rodents) regulate a variety of processes throughout the body. They act on target cells by specific receptors, the high-affinity mineralocorticoid receptor (MR), and the lower-affinity glucocorticoid receptor (GR). Both receptors primarily remain in the cytoplasm as part of multiprotein complexes (containing chaperone proteins such as Heat shock protein 90 (Hsp90), and immunophilins), whereas upon ligand binding receptors translocate into the nucleus to act as transcription factors [22,23]. In addition, the availability of glucocorticoids in the tissues is regulated by a locally expressed specific enzyme, 11β-hydroxysteroid dehydrogenase (11β-HSD) [24,25]. Further complexity in GR/MR signaling is accomplished through different glucocorticoid-response elements (GREs) and multiple receptors isoforms, as well as many post-translational modifications, all able to substantially alter the final functional outcome [25,26].

The synthesis and release of glucocorticoids are under dynamic circadian and ultradian regulation mediated by the hypothalamic-pituitary-adrenal (HPA) axis, also involved in the response to stressful events. By negative feedback regulation, elevated glucocorticoids inhibit their production mainly at the level of the pituitary, with the hippocampus, amygdala, and frontal cortex involved as higher limbic brain regions mediating additional cognitive and/or emotional manifestations in case of stress response [27,28,29]. Glucocorticoid negative feedback mechanisms are mostly mediated by GR, abundantly present in these structures, and activated with higher concentrations of glucocorticoids [30]. Following prolonged stress, however, maladaptive changes occur that lead to further progressive loss of HPA axis control and hypersecretion of glucocorticoids. According to crucial glucocorticoid cascade hypothesis [21], attenuated glucocorticoid feedback efficacy in aging is due to reduced GR expression, translocation, and binding [31,32,33,34], possibly in an attempt to reduce potentially damaging effects of glucocorticoids.

Aging is normally accompanied by a gradual and progressive cognitive decline. Nevertheless, some individuals experience more significant impairments than others [35], and in animal studies, equivalent variability was directly correlated with endogenous activity of HPA axis [36]. The majority of age-related studies were, however, focused on the role of dysregulated GR signaling in impaired spatial memory and structural changes in the hippocampus [31,32,37,38], with just a few studies addressing relevant alterations in the cortex, brain region highly vulnerable to the effects of stress and aging and involved in cognition and regulation of the HPA axis [27,28,29,33]. The effects of FR on HPA axis function were not fully revealed as well. Dose-response relationship between FR and increased corticosterone levels was demonstrated in rats [39], and HPA axis responsiveness to stress was also shown to remain relatively unaffected [40]. So far, the effect of FR on glucocorticoid receptor following short-term every other day feeding paradigm in adult rats revealed a minor compensatory decrease in the level of GR protein, with no change in its mRNA or MR [41]. Therefore, the aim of the present study was to provide a broad analysis of the effects of food restriction on cortical responsiveness to glucocorticoids, relevant changes in glucocorticoid hormone/glucocorticoid receptor (GR) system, and moreover to reveal the pattern of these changes during aging in order to identify their involvement in age-related cognitive decline and numerous well-known neuroprotective effects of FR.

## 2. Materials and Methods

### 2.1. Animals and Feeding Regimen

A total of 30 male Wistar rats were included in the study. Animals were obtained from the animal facility at the Institute for Biological Research, University of Belgrade, and housed under temperature-, humidity-, and light-controlled conditions (23 ± 2 °C, 60–70%, 12-h light/dark cycles), with standard laboratory chow pellets (Veterinarski zavod Subotica, Serbia) and water available ad libitum (AL) until 6 months of age. At that time, average daily food consumption was determined for 7 days and rats were randomly divided into two groups. The AL group continued to receive food ad libitum, whereas the FR group was fed with 100% of the mean daily intake of the AL animals every second day, shortly before the onset of dark cycle. The amount of food for the FR group was readjusted every month. The animals were maintained on established feeding regimens until 18 and 24 months of age and were subsequently euthanized after 12 h of fasting (between 9:00 h and 12:00 h). Brains were quickly extracted, and cerebral cortices were dissected on ice, snap-frozen in liquid nitrogen, and stored at −80 °C for subsequent analysis.

### 2.2. Corticosterone Assay

The cortical corticosterone concentrations were determined using competitive enzyme immunoassay (Corticosterone High Sensitivity EIA kit; IDS, East Boldon, UK) according to the manufacturer’s instructions, and as previously described [42,43]. In brief, corticosterone in tissue homogenates (in five volumes of 1×PBS) was extracted three times with diethyl ether and combined organic phases were subsequently evaporated and diluted in the assay buffer. Standards and samples were measured in duplicates at 450 nm and 650 nm using WALLAC 1420-Victor 2 Multilabel Counter (PerkinElmer, Waltham, MA, USA). The coefficient of variance among the duplicates was <7%. For plotting the standard curve and data extrapolation 4PL curve fitting method (GraphPad Prism, v. 5; GraphPad Software Inc., San Diego, CA, USA) was used. The hormone concentrations are expressed as ng/g of tissue.

### 2.3. RT-PCR

Total RNA was isolated from the cerebral cortices using Trizol reagent (Invitrogen/Thermo Fisher Scientific, Waltham, MA, USA) according to the manufacturer’s instructions. One microgram of RNA was treated with 1 U of RNase-free DNase I (Thermo Fisher Scientific, Waltham, MA, USA) and reverse transcribed using a High-Capacity cDNA Archive Kit (random hexamer primers;Applied Biosystems/Thermo Fisher Scientific, Waltham, MA, USA). The reactions were performed at 25 °C for 10 min and then at 37 °C for 2 h, in a final volume of 10 μL.

Real-time PCR analysis for *11βhsd*-1, *gfap,* and *sgk-1* mRNAs was performed using commercially available rat Assays-on-Demand Gene Expression Products (Applied Biosystems/Thermo Fisher Scientific, Waltham, MA, USA). The reaction mixtures with TaqMan Universal Master Mix containing AmpErase UNG (Applied Biosystems/Thermo Fisher Scientific, Waltham, MA, USA), Assay Mix (Applied Biosystems/Thermo Fisher Scientific, Waltham, MA, USA), and cDNA templates (conversion of 1 ng of RNAs) were amplified in the ABI Prism 7000 Sequence Detection System at 50 °C for 2 min, 95 °C for 10 min, followed by 40 cycles of 95 °C for 15 s, and at 60 °C for 1 min. Validation experiments were performed as previously described [44], with glyceraldehyde 3-phosphate dehydrogenase (GAPDH) or β-actin selected as reference genes (rat Assays-on-Demand Gene Expression Products; Applied Biosystems/Thermo Fisher Scientific, Waltham, MA, USA). Analysis was performed by RQ Study Add ON v 1.1 software (Applied Biosystems/Thermo Fisher Scientific, Waltham, MA, USA) with a confidence level of 95% (*p* < 0.05). Final results are shown as the fold changes relative to the value of the 6-month-old rat sample that was assigned the value of 1.

*Mr*, *gr* (*nr3c1*—Nuclear Receptor Subfamily 3 Group C Member 1), *bax* and c-*fos* mRNA levels were examined by semiquantitative RT-PCR analysis in a total volume of 25 μL containing 150 ng cDNA, 1.5 mM MgCl2, 0.2 mM dNTPs and 1 U of Taq polymerase (Fermentas/Thermo Fisher Scientific, Waltham, MA, USA). The primer sequences were as follows (forward primer/reverse primer-5′-3′): MR-AGCTCTTCTGTTAGCAGCCCGCTG /CTGAAGTGGCATAGCTGAAGGCAT; GR-TGCAAACCTCAATAGGTCGACCAG/TAAACTGGGCCCAGTTTCTCTTGC; Bax-GGCGAATTGGAGATGAACTG/TTCTTCCAGATGGTGAGCGA; c-fos-GACCGAGATTGCCAATCTAC/GGAAACAAGAAGTCATCAAAGG; GAPDH-GGAGTCAACGGATTTGGTCGTAT/AGCCTTCTCCATGGTGGTGAAGAC; β-actin-TGGACATCCGCAAAGACCTGTAC/TCAGGAGGAGCAATGATCTTGA. All RT-PCR reactions were performed in triplicate and two independent RT reactions, in the GeneAmp1 PCR System 9700 (Applied Biosystems/Thermo Fisher Scientific, Waltham, MA, USA), starting at 95 °C for 2 min, followed by 31 cycles at 95 °C for 15 s, at 58 °C for 30 s and at 72 °C for 30 s. Final elongation was performed at 72 °C for 5 min. The PCR products were visualized by ethidium bromide staining of 2% agarose gels. For densitometric analysis, a Multi-Analyst/PC Software Image Analysis System (Gel Doc 1000; Bio-Rad, Hercules, CA, USA) was used. Relative mRNA levels are expressed as the fold change relative to data obtained in 6-month-old animals that were assigned the value of 1 (*n* = 6).

### 2.4. Immunoblotting and Antibodies

For whole cell lysates, cortical tissue was homogenized and sonicated in 10 volumes of RIPA buffer (50 mM Tris–HCl, pH 7.5, 150 mM NaCl, 0.1% SDS, 1% NP-40, 0.5% Triton X-100) containing protease and phosphatase inhibitors (Roche Diagnostics, Basel, Switzerland). For cytoplasmic and nuclear fractions, a commercially available kit was used (NE-PER™ Nuclear and Cytoplasmic Extraction Reagents, Thermo Scientific, Waltham, MA, USA), according to the manufacturer’s instructions. Following the centrifugation at 20,800× *g* at 4 °C for 30 min, protein concentration was determined using the Micro BCA Protein Assay Kit (Pierce Biotechnology, MA, USA). Protein extracts (20 μg per lane) were further separated by SDS-polyacrylamide gel electrophoresis and blotted onto PVDF membranes (GE Healthcare, Little Chalfont, UK). The membranes were then incubated for 1 h at room temperature in 5% non-fat dry milk/TBST (150 mM NaCl, 50 mM Tris, pH 7.4, and 0.1% Tween 20) to block non-specific binding. The following primary antibodies were used: rabbit anti-GR (P-20, Santa Cruz Biotechnology, Dallas, TX, USA), rabbit anti-pSer^211^ GR (Cell Signaling Technology, Danvers, MA, USA), goat anti-11β-HSD1 (R&D Systems, Minneapolis, USA), rabbit anti-CDK5 (C-8, Santa Cruz Biotechnology, Dallas, TX, USA), rabbit anti-p35 (C-19, Santa Cruz Biotechnology, Dallas, TX, USA), rabbit anti-ERK (Millipore, Sigma, St. Louis, MO, USA), mouse anti-pTyr^204^ ERK (E-4, Santa Cruz Biotechnology, Dallas, TX, USA), rabbit anti-FKB51 (H-100, Santa Cruz Biotechnology, Dallas, TX, USA), mouse anti-HSP90 (F-8, Santa Cruz Biotechnology, Dallas, TX, USA), and rabbit anti-NFκB (p65; C-20, Santa Cruz Biotechnology, Dallas, TX, USA). Following several rinses in TBST, the membranes were incubated for 1 h in appropriate Horseradish peroxidase (HRP)-(Santa Cruz Biotechnology, Dallas, TX, USA), or Alexa-488-conjugated secondary antibody (Invitrogen/Thermo Fisher Scientific, Waltham, MA, USA). HRP-immunoreactive bands were visualized using an enhanced chemiluminiscence kit (ECL, GE Healthcare, Little Chalfont, UK) and film exposure, while for fluorescent signals, STORM system for non-radioactive detection (GE Healthcare, Little Chalfont, UK) was used. Each blot was subsequently re-probed with goat anti-GAPDH (Santa Cruz Biotechnology, Dallas, TX, USA) or rabbit anti-β-actin antibody (Millipore, Sigma, St. Louis, MO, USA). The signals were quantified using Image Quant software (v. 5.2, GE Healthcare, Little Chalfont, UK) and expressed as relative values normalized to the corresponding GAPDH or β-actin signals. For phosphorylated proteins, the levels were further normalized to the level of total proteins. The expression of the target proteins in each experimental group (AL and FR) was determined as the fold change relative to the appropriate controls that were assigned the value 1 (*n* = 6).

### 2.5. Statistical Analysis

All values are shown as the mean ± SEM. Statistical analysis was performed using Statistica 6.0 software (StatSoft Inc., Tulsa, OK, USA). The normality of data sets was estimated by the Shapiro–Wilk’s test. Consequently, the differences between the experimental groups were tested using the nonparametric Mann–Whitney U Test. For the effect of aging, values were calculated relative to the 6-month-old rat sample, and for the effects of food restriction relative to age-matched controls. Significance was set at *p* < 0.05.

## 3. Results

### 3.1. Food Restriction Increases Cortical Levels of Corticosterone and 11β-HSD1 Protein

**Figure 1:**
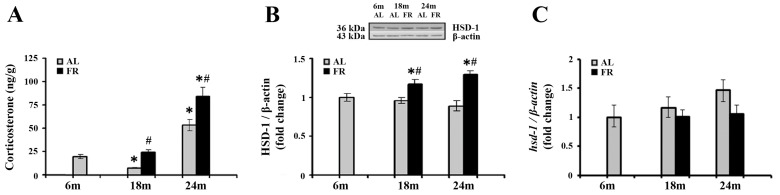
The effect of food restriction on corticosterone and 11β-hydroxysteroid dehydrogenase 1 (11β-HSD1) levels in the rat cortex during aging. Basal tissue corticosterone concentration (**A**), relative 11β-HSD1 protein level (**B**), and 11β-HSD1 mRNA level (**C**) were determined in rats fed ad libitum (AL) (gray bars) or subjected to food restriction (FR) (black bars). Relative 11β-HSD1 protein and mRNA levels are presented as the mean ± SEM of fold changes relative to the values obtained in control, 6-month-old AL animals (*n* = 6). * *p* < 0.05 vs. 6-month-old rats; # *p* < 0.05 vs. age-matched AL rats.

Numerous studies reported that aging and various FR regimens are accompanied by an increase in blood levels of glucocorticoids, while the relevant changes in cortical tissue were not examined so far. The analysis of corticosterone levels in our experimental paradigm revealed alterations in the cortex, both during aging and following food restriction (Figure 1A). Namely, subsequent to transient decline in 18-month-old AL rats (* *p* < 0.05), cortical corticosterone level was significantly increased in 24-month-old rats in comparison to the levels estimated in control, 6-month-old rats (by 2.7 fold; * *p* < 0.05). Relative to the same control, food restriction-induced further increase in the level of corticosterone only in 24-month-old FR rats (by 4.3 fold; * *p* < 0.05). However, in comparison to AL age-matched controls, increased hormone level was detected in both age groups examined. In 18-month-old rats this increase was 3.1-fold, and in 24-month-old rats by 58% (Figure 1A; # *p* < 0.05).

We next examined how detected increase in cortical corticosterone levels correlated with the expression of 11β-HSD1, the enzyme that regenerates the active glucocorticoids from inert 11-keto forms in brain cells, and have an impact on the GR activity through ligand availability [24]. The results obtained by Western blot analysis (Figure 1B) revealed that protein levels of 11β-HSD1 did not change during aging, while food restriction significantly increased the level of 11β-HSD1 in both age groups examined in comparison to the age-matched AL animals (in 18-month-old rats by 21% and 24-month-old rats by 45%; *p* < 0.05). In contrast, mRNA levels of 11β-HSD1 did not change following food restriction (Figure 1C), and a trend toward the increase during aging was observed, in line with data reported previously [45].

### 3.2. The Effects of Aging and Food Restriction on Glucocorticoid Receptor in the Cortex

**Figure 2:**
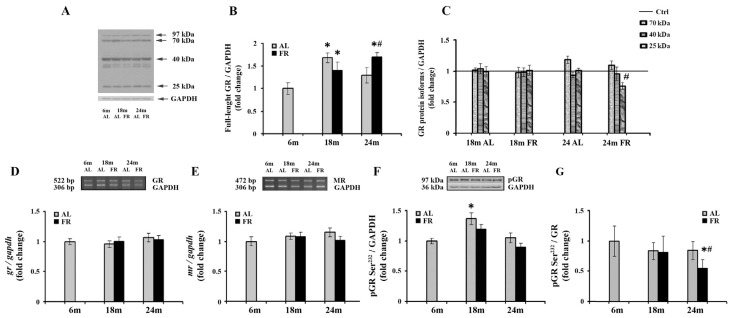
The effect of food restriction on the levels of glucocorticoid and mineralocorticoid receptors in the rat cortex during aging. (**A**) Representative blot with full-length glucocorticoid receptor protein and shorter GRα protein isoforms (molecular weights indicated). Relative protein levels of the full-length glucocorticoid receptor (**B**), 70 kDa, 40 kDa, and 25 kDa GR isoforms additionally normalized to the values obtained in control (Ctrl.), 6-month-old animals presented as the line (**C**), GR mRNA (**D**), mineralocorticoid receptor (MR) mRNA (**E**), and GR phosphorylated at Ser^232^ (pGR) (**F**) were determined in rats fed ad libitum (AL) (gray bars) or subjected to food restriction (FR) (black bars). (**G**) The level of GR phosphorylated at Ser^232^ relative to the level of total GR. Data are presented as the mean ± SEM of fold changes relative to the values obtained in control, 6-month-old AL animals (*n* = 6). * *p* < 0.05 vs. 6-month-old rats; # *p* < 0.05 vs. age-matched AL rats.

Given the involvement of glucocorticoid receptor in the complex HPA axis regulation and age-related deficient glucocorticoid feedback efficacy, we continued with a comprehensive analysis of the GR expression following long-term food restriction in cortical tissue (Figure 2). The expression profiles of the GRα protein isoforms generated as a result of alternate translation start sites in exon 2 and contributing to complex regulation by glucocorticoids were also performed [46]. As expected, besides the full-length glucocorticoid receptor (97 kDa GR), shorter GRα isoforms (67-, 50-, 40-, and 25-kDa) were detected in rat cortical tissue (Figure 2A), with the 40 kDa isoform being the most prevalent one, as previously reported [43,47]. Quantitative immunoblot analysis also revealed an increase of full-length glucocorticoid receptor (97 kDa GR) in 18-month-old AL rats (68%; * *p* < 0.05), while in the 24-month-old rats the level of 97 kDa GR was not significantly altered in relation to the level estimated in control, 6-month-old rats (Figure 2B). Food restriction also induced an increase in the level of 97 kDa GR. In 18-month-old animals, GR protein levels were increased by 40% (* *p* < 0.05), while in 24-month-old FR rats the increase was by 70% and also significantly higher than in AL age-matched control (by 32%; # *p* < 0.05). Among GR translational isoforms, only the isoform of 25 kDa was altered in aged animals following EOD feeding. Other isoforms, although significantly increased in comparison to full-length GR, were not changed relative to their values in 6-month-old animals, regardless of the feeding regimen applied (Figure 2C). The decrease of 25 kDa isoform in aged animals subjected to FR was by 27% and was in line with an observed increase in full-length protein. Finally, RT-PCR analysis revealed that the level of GR mRNA did not change during aging, regardless of the feeding regimen applied (Figure 2D). Similarly, in our experimental paradigm no change in MR mRNA levels was detected (Figure 2E).

Despite the observed increase in full-length GR, long-term FR did not affect the level of GR phosphorylated at Ser^232^ (pGR) (Figure 2F). In both age groups examined, the levels of pGR were not significantly different from the levels determined for 6-month-old rats and age-matched controls. No consistent impact of aging on the level of pGR throughout the overall follow-up period was detected as well. In 18-month-old AL animals, a transient increase that corresponded to the increase observed for the full-length protein in the same experimental group versus 6-month-old rats was obvious (Figure 2B; 37%; * *p* < 0.05). Again, as for full-length GR, in old rats, the levels returned at the same level observed in control, 6-month-old animals. The level of pGR relative to the level of total GR was however significantly decreased in 24-month-old FR animals due to the increase in full-length GR, and thus clearly implying the decrease in its phosphorylation efficiency (Figure 2G; 45%; # *p* < 0.05).

### 3.3. Food Restriction Have no Effect on CDK5 Protein Level, but Alters Hsp90, and FKBP51 in the Cortex during Aging

**Figure 3:**
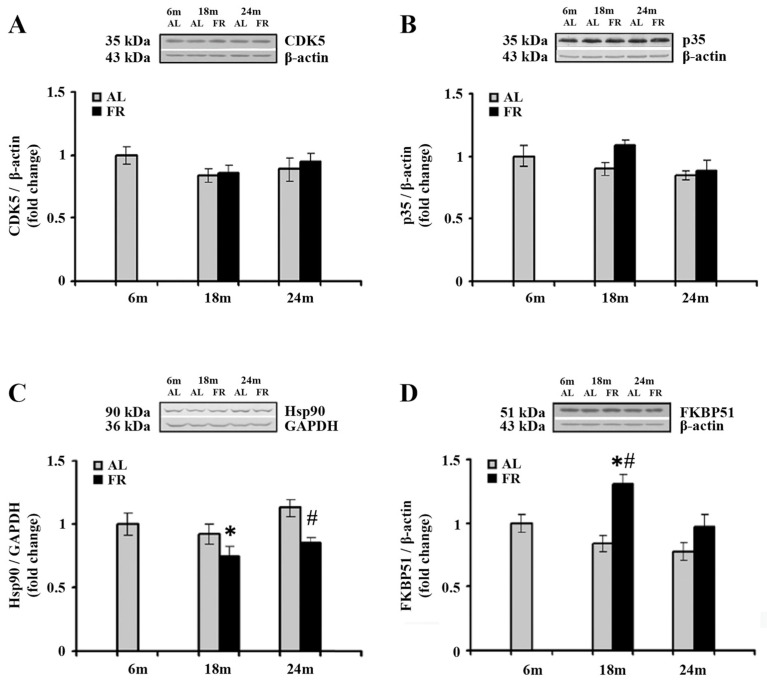
The effect of food restriction on the levels of cyclin-dependent kinase 5 (CDK5), its activator, p35, Hsp90, and FKBP51 levels in the rat cortex during aging. Relative protein levels of the CDK5 (**A**), p35 (**B**), Hsp90 (**C**), and FKBP51 (**D**) were determined by Western blot analysis in rats fed ad libitum (AL) (gray bars) or subjected to food restriction (FR) (black bars). Data are presented as the mean ± SEM of fold changes relative to the values obtained in control, 6-month-old AL animals (*n* = 6). * *p* < 0.05 vs. 6-month-old rats; # *p* < 0.05 vs. age-matched AL rats.

Cyclin-dependent kinase 5 (CDK5) phosphorylates GR at Ser^232^ [48], in association with its major neuron-specific activator, protein, p35. The impact of aging and food restriction on the expression of CDK5 and p35 was examined by Western blot analysis, and the results are presented in Figure 3. In the cortical tissues obtained from all experimental group examined, there was no statistically significant difference in the level of both CDK5 and p35 (Figure 3A,B).

EOD feeding-induced increase in cortical GR, prompted us to further analyze the expression profiles of two protein members of the GR complex as the proteins essential for several intracellular GR signaling pathways in the cytoplasm and their translocation into the nucleus. The Western blot analysis of Hsp90, as well as the analysis of immunophilin FK506 binding protein 51 (FKBP51) revealed alterations in the cortex of EOD-fed rats (Figure 3C,D). Namely, a significant decrease in Hsp90 was detected only in 18-month-old EOD rats when compared to the 6-month-old control (Figure 3C; 25%; * *p* < 0.05), whereas in 24-month-old rats food restriction decreased the level of Hsp90 relative to the level detected in AL age-matched control (Figure 3C; 25%; # *p* < 0.05). In addition, food restriction increased the level of FKBP51 in 18-month-old rats when compared to the AL age-matched and 6-month-old animals (Figure 3D; 55% and 30%; respectively; *p* < 0.05), while in aged animals its levels remained unchanged, implying normalization in the level of this immunophilin.

### 3.4. Food Restriction Reverted Age-Related Increase in Gfap and Bax mRNA Level and Induced c-fos mRNA Increase

**Figure 4:**
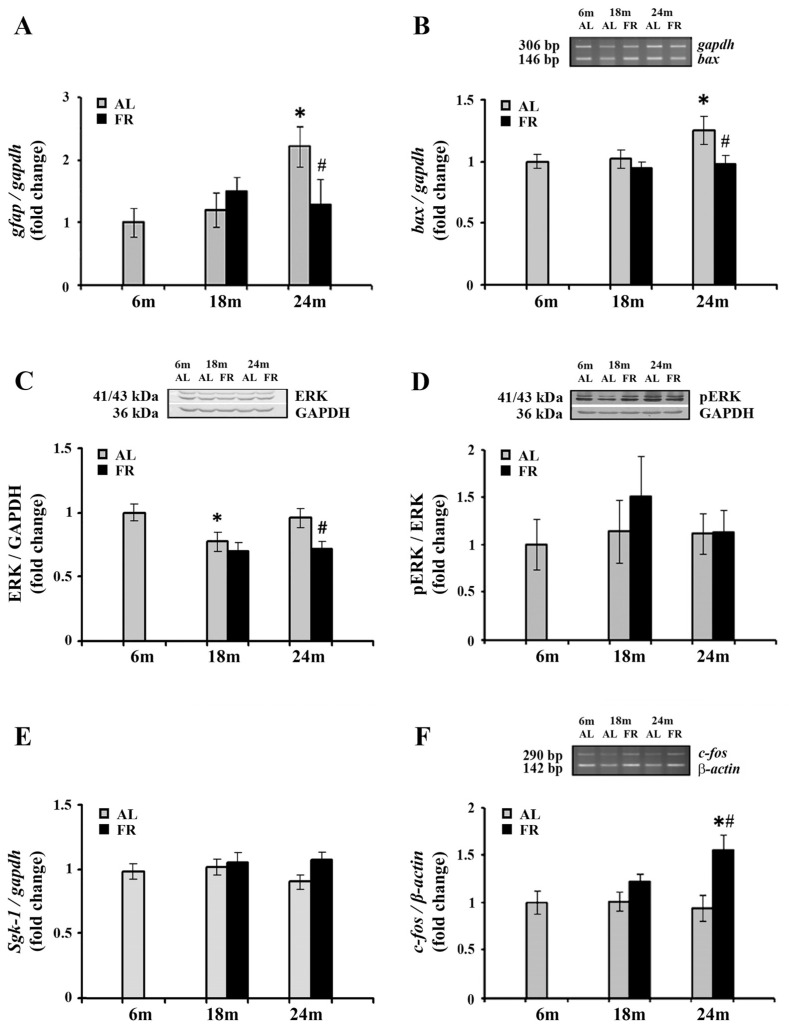
The effect of food restriction on the neuronal inflammation and activity in the rat cortex during aging. The levels of *gfap* (**A**), *bax* (**B**), *sgk-1* (**E**) and *c-fos* mRNAs (**F**) were determined by quantitative RT-PCR, while relative protein levels of ERK (**C**) and pERK (**D**) were determined by Western blot, in rats fed ad libitum (AL) (gray bars) or subjected to food restriction (FR) (black bars). Data are presented as the mean ± SEM of fold changes relative to the values obtained in control, 6-month-old AL animals (*n* = 6). * *p* < 0.05 vs. 6-month-old rats; # *p* < 0.05 vs. age-matched AL rats.

We next analyzed mRNA level of glial fibrillary acidic protein (GFAP) in the cortex of aging rats (Figure 4). As expected, a significant increase in *gfap* mRNA was detected in aged, 24-month-old AL rats (by 2.2 fold; * *p* < 0.05). Moreover, in line with the well-known anti-inflammatory effects of FR, food restriction counteracted this age-related increase reverting the expression of *gfap* mRNA to the level obtained for 6-month-old control rats (Figure 4A). In comparison to age-matched control, the level of *gfap* mRNA was decreased by 43% (# *p* < 0.05).

The known anti-apoptotic effects of FR were also confirmed in our experimental paradigm. Aging induced an increase in mRNA expression of Bcl-2-associated X protein (Bax) (Figure 4B), a pro-apoptotic member of the Bcl-2 gene family that is considered as the canonical membrane permeabilizing effector. In 24-month-old rats, however, long-term food restriction-induced the decrease in its RNA level by 25% (# *p* < 0.05), reaching the level detected in control, 6-month old rats.

On the other hand, Western blot analysis of extracellular signal-regulated kinase 1/2 (ERK1/2), with an important role in neuronal apoptosis under conditions of stress [49,50] revealed its decrease in the cortex of 24-month-old EOD-fed rats, by 25% (Figure 4C; # *p* < 0.05). The level of ERK1/2 phosphorylated at Thr^202^/Tyr^204^ was similarly decreased, by 27%, in comparison to 6-month-old control rats and age-matched controls. The relative ratio of pERK and ERK revealed therefore no changes in all agegroups examined (Figure 4D).

Expression levels of one of the canonical GR targets, serum- and glucocorticoid inducible kinase 1 (Sgk-1), that has tandem GREs located approximately 1 kb upstream of its transcription start site, were further analyzed by qPCR (Figure 4E). The increase in corticosterone following FR was not however sufficient to alter transcription of this particular GR target gene. Namely, the level of *sgk-1* mRNA was not changed neither during aging nor following long-term food restriction in all age groups examined.

Finally, we examined the expression c-Fos, widely used as a functional marker of neuronal activity following a variety of stimuli [51,52,53]. RT-PCR analysis revealed that aging did not affect *c-fos* gene expression (Figure 4E). However, in the cortex of 24-month-old animals, EOD induced a significant increase *c-fos* mRNA. The level of *c-fos* mRNA was increased by more than 55% compared to both the 6-month-old and age-matched AL controls, while no changes were detected in 18-month-old EOD-fed animals (Figure 4E; # *p* < 0.05).

### 3.5. Food Restriction Reverted the Alterations in NFκB Intracellular Distribution

**Figure 5:**
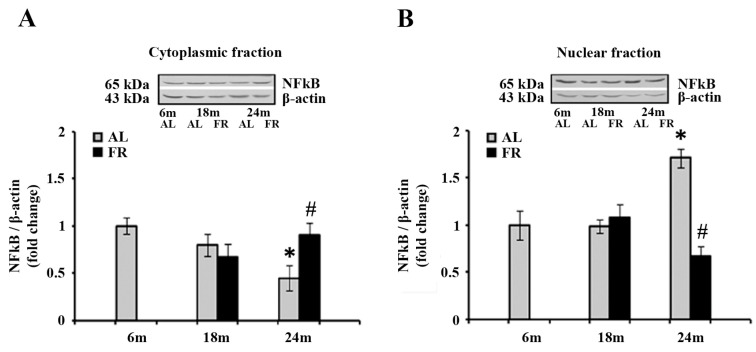
The effect of food restriction on the nuclear factor-kappa B (NFκB) abundance in the rat cortex during aging. Relative NFκB protein levels in the cytoplasmic fraction (**A**) and nuclear fraction (**B**) were determined in rats fed ad libitum (AL) (gray bars) or subjected to food restriction (FR) (black bars). Data are presented as the mean ± SEM of fold changes relative to the values obtained in control, 6-month-old AL animals (*n* = 6). * *p* < 0.05 vs. 6-month-old rats; # *p* < 0.05 vs. age-matched AL rats.

Considering that the food restriction reverted the increase in *gfap* mRNA level in aged AL rats and had no effect on the level of pGR, we next examined the intracellular distributions of the additional transcription factor, nuclear factor-kappa B (NFκB) (Figure 5).

Analysis of the NFκB by Western blot revealed anaging-induced decrease in the level of NFκB in the cytoplasm (Figure 5A), and translocation of this transcription factor to the nucleus (Figure 5B). Namely, the level detected in the cytoplasmic fraction of 24-month-old AL rats was 55% lower than in the 6-month-old control (Figure 5A; * *p* < 0.05), whereas the level detected in the nuclear fraction, was 71% higher in the same experimental groups (Figure 5B; * *p* < 0.05). On the other hand, food restriction neutralized these changes, reverting the expression of NFκB to the level measured in 6-month-old control rats in both cytoplasmic and nuclear fractions (Figure 5).

## 4. Discussion

The results of the present study demonstrate an increase in the cortical level of corticosterone during aging in rats and its further increase in animals on EOD-feeding regimen. 11β-HSD1, a key enzyme in brain tissue that determines the availability of corticosterone to its receptors, was, however, increased only in the cortex of rats subjected to long-term food restriction. We also detected an increase in the cortical level of glucocorticoid receptor following food restriction in aged animals, without alteration in pGR level. Unaltered pGR level was in line with no changes in CDK5 level, together with the decrease in the level of Hsp90 and an increase in a negative regulator of GR function, FKBP51. Moreover, our data revealed age-induced translocation of transcription factor NFκB to the nucleus, whereas food restriction reverted these changes, restoring its nuclear and cytoplasmic abundance to the level observed in 6-month-old control rats. Finally, reduced food intake prevented an age-related increase in the cortical level of GFAP and Bax, confirming its anti-inflammatory and anti-apoptotic effects, whereas the increase in the expression of *c-fos* implies that food restriction tends to maintain the reactivity of cortical neurons during aging.

Elevation in basal plasma corticosterone levels during aging, and following various types of food restriction regimens is well documented [13,14,15,20,21]. Age-related increase in corticosterone was associated with impaired HPA negative-feedback regulation, and further with cognitive deficits, while elevated circulating glucocorticoids in FR are in contrast to its well accepted protective effects on neurons and synapses [3,4,5,9]. Namely, deleterious effects of excessive and prolonged elevation in glucocorticoids are well known to involve structural and functional alterations that lead to impairments in brain functions and HPA activity [21,31,32,37,38]. In regard to the complex regulation of glucocorticoids effects in different tissues including the brain [25], and postulated “glucocorticoid paradox” of FR, i.e., its protective effects despite the increase in corticosterone that can be detrimental [54], the present study is an attempt to reveal the causative role of glucocorticoid hormone/GR system in such contrasting effects. A similar paradox was reported for exercise-induced neuroprotective effects accompanied by the rise of glucocorticoids as well [55].

The corticosterone level within the tissue depends on plasma corticosterone level as well as on the local intracellular metabolism and its regeneration from inert 11-keto forms by 11β-HSD1 [24]. We have shown for the first time that the level of corticosterone in the cortex directly reflects the pattern reported for plasma corticosterone both during aging and following FR [13,14,15,20] and that 11β-HSD1 may be involved in more pronounced FR-induced corticosterone increases. In the adult brain, 11β-HSD1 is expressed particularly in the hippocampus, cortex, and amygdala, and an age-related increase in its mRNA level has been correlated to 11β-HSD1 activity and cognitive decline [45,56]. Although it was shown that mice lacking *11β-hsd* are protected from age-related cognitive deficits [57], these mice also exhibited increased basal HPA activity and its reduced sensitivity to glucocorticoid negative feedback in response to stress [58]. 11β-HSD1 appeared therefore as the critical regulator in proper HPA functioning. This is further in line with our results revealing an age-related increase in 11β-HSD1 mRNA with a trend toward the decrease in protein level that was reverted by EOD feeding, indicating further normalization of HPA reactivity following EOD feeding.

Despite elevated corticosterone levels, anticipated ligand-induced down-regulation of cortical glucocorticoid receptors was not observed in EOD-fed animals. Furthermore, in contrast to our previous results for the hippocampus, where the decrease in GR protein level was obvious and accompanied with EOD-induced increase in *mr MRNA* during aging [43], no change in both *gr* and *mr* mRNA levels was observed in the cortex following long-term FR. The latter is particularly expected, given that MR is less abundant in this brain structure [30]. The diverse cortical and hippocampal response was also reported in adult rats subjected to a short-term EOD regimen, where no changes in cortical MR and a reduction of GR by 20% was reported solely at the protein level [41]. On the other hand, overexpression of GR protein in mice has also been associated with enhanced HPA system feedback [59], implying EOD-induced improvements in feedback inhibition of the HPA axis and glucocorticoid resistance associated with aging at this level as well.

Regardless of the increased level observed in EOD animals, our data indicate that cortical GRs were not, however, all transcriptionally active. The level of GR phosphorylated at Ser^232^ (pGR) that has been associated with increased transcriptional activity of GR [26] was not changed, in spite of the observed increase in relevant ligand, corticosterone. This further implied improper GR functioning that has been proposed to be correlated to its altered ligand affinity, translocation to the nucleus, DNA binding, or interaction with other transcription factors [21,31,32,33,34]. In that regard, attenuated ligand affinity and nuclear translocation have been ascribed to the reassociation with chaperones and immunophilins involved in maturation, degradation, and nuclear transport of GR like Hsp90 and FKBP51 [22,60,61].

Well described anti-inflammatory effects of FR [62,63,64], reflected here by the decrease of GFAP level in old animals, also points toward the predominance of another well-known transcriptional mechanism of GR, transrepression. Indeed, glucocorticoids are widely used in clinical practice as immunosuppressive agents considering that GR physically interacts, usually as a monomer, and interfere with the activity of pro-inflammatory transcription factors like NFκB [65]. Our results for NFκB also provided evidence that food restriction may decrease inflammation by suppressing its signaling, in line with other studies [66,67]. Furthermore, besides numerous GR polymorphisms and mutations that were linked with its context-specific transcriptional regulation and glucocorticoid resistance in humans, GR mRNA alternative splicing and alternate translation initiation were shown to cause expression of GR isoforms with different transcriptional activity [46]. In aged EOD-fed animals, our analyses indicated the involvement of short, 25-kDa GRα isoform that was decreased in parallel with an increase in full-length GRα. Although the function of this particular isoform has not been fully revealed, it was demonstrated that GRα translational isoforms lacking the entire AF1 transactivation domain are constitutively expressed in the nucleus, and are with limited capacity to induce transcriptional activity [46]. In the same study, it was also revealed that capacity to bind NFκB p65 was, however, preserved and equivalent to the capacity of GRα in cell-free systems [68]. In addition, it was further found that the short translational isoform failed in full antagonism of NFκB in cells [69] probably since cytoplasmic colocalization is required for optimal association of GR with p65 [70]. Altered expression of GRα isoforms was associated with glucocorticoid resistance [69], psychiatric disorders, and hyporesponsiveness of the neonatal HPA axis in humans [47,71]. The decrease in short isoform, therefore, implies the restoration of GR anti-inflammatory effects following EOD feeding, and its associated anti-apoptotic effects, reflected by the decrease of Bax in aged animals. In line with this, ERK-mediated signaling that plays an important role in neuronal apoptosis under conditions of stress [49,50] was also decreased. The increase in the expression of *c-fos* also indicates that food restriction during aging tends to maintain the reactivity of cortical neurons and participates in the mechanism facilitating HPA function during stress [72].

In conclusion, the data presented here confirm and expand the finding that long-term FR affects cortical responsiveness to glucocorticoids, emphasizing that this effect is more pronounced in aged animals. Moreover, the present study provides additional insight into molecular mechanisms of food restriction-induced cortical remodeling involving glucocorticoid receptors that are of importance for proper HPA functioning and final behavioral outcomes. In context of well-known attenuation of age-related inflammation, impaired synaptic plasticity, and cognitive decline that is commonly induced by FR, the present study contributes to further search for potential therapeutic targets and general recommendations for behavioral habits that will assure a long and healthy life in humans.
